# Sorbicillinoids hyperproduction without affecting the cellulosic enzyme production in *Trichoderma reesei* JNTR5

**DOI:** 10.1186/s13068-022-02183-1

**Published:** 2022-08-22

**Authors:** Chengcheng Li, Ruihan Gu, Fengming Lin, Huining Xiao

**Affiliations:** 1 School of Light Ind. & Food Sci. and Jiangsu Co-Innovation Center for Efficient Processing and Utilization of Forest Resources and International Innovation Center for Forest Chemicals and Materials, Nanjing Forestry University, Nanjing, 210037 China; 2 State Key Laboratory of Bioelectronics, School of Biological Science and Medical Engineering, Southeast University, Nanjing, 210096 China; 3 Department of Chemical Engineering, University of New Brunswick, Fredericton, NB E3B 5A3 Canada

**Keywords:** *Tr69957*, *Trichoderma reesei*, Yellow pigment, Sorbicillinoids, Secondary metabolites, Hyperproduction

## Abstract

**Background:**

Microbial production of bioactive secondary metabolites is challenging as most of the encoding genes are silent; and even if they are activated, the biosynthetic pathways are usually complex. Sorbicillinoids with multifunctional bioactivities are examples of these problems, which if solved can result in a more sustainable, simple supply of these important compounds to the pharmaceutical industry. As an excellent producer of cellulosic enzymes, *Trichoderma reesei* can secrete various sorbicillinoids.

**Results:**

Here, we obtained a *T. reesei* mutant strain JNTR5 from the random mutation during overexpression of gene *Tr69957* in *T. reesei* RUT-C30. JNTR5 exhibited a significant constitutive increase in sorbicillinoids production without affecting the cellulosic enzyme production. Confocal laser scanning microscope (CLSM) results indicated that sorbicillinoids were distributed in both mycelium and spores of JNTR5 with blue and green fluorescence. Compared with RUT-C30, JNTR5 displayed different cell morphology, reduced growth rate, and increased sporulation, but a similar biomass accumulation. Furthermore, transcriptome analysis revealed that all genes belonging to the sorbicillinoid gene cluster were upregulated, while most cellulase-encoding genes were downregulated. The cell wall integrity of JNTR5 was damaged, which might benefit the cellulase secretion and contribute to the almost unchanged cellulase and hemicellulase activity given that the damaged cell wall can enhance the secretion of the enzymes.

**Conclusions:**

For the first time, we constructed a sorbicillinoids hyperproduction *T. reesei* platform with comparable cellulosic enzymes production. This outperformance of JNTR5, which is strain-specific, is proposed to be attributed to the overexpression of gene Tr69957, causing the chromosome remodeling and subsequently changing the cell morphology, structure, and the global gene expression as shown by phenotype and the transcriptome analysis of JNTR5. Overall, JNTR5 shows great potential for industrial microbial production of sorbicillinoids from cellulose and serves as an excellent model for investigating the distribution and secretion of yellow pigments in *T. reesei*.

**Supplementary Information:**

The online version contains supplementary material available at 10.1186/s13068-022-02183-1.

## Background

Fungi are known to be able to secrete a large range of secondary metabolites (SMs) with high bioactivity. SMs are not essential for survival of the producer, but provide competitive advantages for environmental adaptation [1]. Most of SMs are derived from either polyketides (PKSs) such as the yellow spore-pigment intermediate naphthopyrone and lovastatin in *A. nidulans* [2, 3], non-ribosomal peptides (NRPs) like the cyclic undecapeptide cyclosporine [4], or mixed. While others come from terpenes or indole alkaloids [5]. Such SMs can expand the living environments of microorganisms or fending off competitors in a given ecological niche, such as penicillin and β-lactam produced by *Penicillium chrysogenum* [6].

Sorbicillinoids that are generally referred to the compounds containing the carbon skeleton of sorbicillin belong to the secondary metabolites of hexaketide derived from PKSs [7]. They are composed by a mixture of different sorbicillin derivatives possessing elaborate circular structure, as well as the C1–C6 sorbyl side chain structure [8]. These sorbicillinoids include more than 90 oxygenated molecules which are divided into four groups [7, 9]. Several genera of ascomycetes can produce sorbicillinoids, including *P. chrysogenum* [10], *Penicillium notatum* [11], *Verticillium intertextum* [12], some *Trichoderma* species [13], *Aspergillus* [14], *Ustilaginoidea virens* [15], and *Streptomyces* [16]. Originally, the yellow pigment is hoped to be eliminated from the culture of *Penicillium* species which are the main producer of β-lactams [6] and *T. reesei* fermentation process when producing cellulase [13]. However, recently, sorbicillinoids are found to possess many bioactivities and pharmaceutical values, including antioxidant [17], anti-HIV [18], and antimicrobial activity [19]. Since Cram et al. first discovered them from *Penicillium notatum* in 1948 [11], extensive studies on finding similar new compounds, structure illustration, and chemical synthesis are continuously proceeding. However, scientists ignored the natural source of sorbicillinoids, and less research was performed on the large-scale microbial production and the regulatory mechanism. The sorbicillinoids hyperproduction strains that can be used for producing sorbicillinoids and serve as the platform for studying the biosynthesis and secretion mechanism are thus urgently needed to be established.

Cellulose conversion for sustainable production of valuable biofuels, chemicals, or materials has attracted more and more interest for relieving the depletion of fossil energy and global warming [20]. Genetically engineered strains have been widespread developed to convert cellulose to fermentable sugars, like *E. coli* [21], *Bacillus methanolicus* [22], and so on. *Trichoderma reesei* is routinely recognized as one of the most important industrially exploited (hemi)cellulase-producing strains [23], well-known for its cellulosic materials degrading ability. Besides, *T. reesei* can also produce the yellow pigment, sorbicillinoids, using cellulose as the carbon source [24, 25]. The biosynthesis pathway of sorbicillinoids in *T. reesei* has been proposed to be organized in one sorbicillinoid gene cluster, including eight genes *sor5*, *sor1*, *sor2*, *sor3*, *sor6*, *ypr2*, *sor4*, and *ypr1*, of which *ypr1* and *ypr2* are two transcription regulators [13, 24, 26, 27]. In addition, the biosynthesis of such yellow pigment is also regulated by XPP1 through regulating a conserved putative secondary metabolism cluster, which contains two PKS encoding genes (TRIREDRAFT_73621 and TRIREDRAFT_73618) essential for the typical yellow pigment production in *T. reesei* [25]*.* The promised chromosome remodeling caused by insertion of gene 121121 deletion cassette may also be responsible for the sorbicillinoids production in *T. reesei* [24]. Although these studies furthered the mechanism study of the sorbicillinoids production, they are still not enough for completely understanding the biosynthesis mechanism of sorbicillinoids and obtaining the sorbicillinoids hyperproducing strain.

In this study, the recombinant *T. reesei* strain JNTR5 was constructed by random insertion of gene Tr69957 in the genome of RUT-C30. Strain JNTR5 showed high-yield sorbicillinoids production without significantly affecting the cellulase and hemicellulase production. The influence of cultivation conditions on sorbicillinoids production, including carbon sources and light was investigated. And the phenotype profiling of JNTR5, such as, biomass accumulation, sporulation, growth rate, cell wall integrity and sorbicillinoids properties were also investigated. Furthermore, RNA-seq was carried out to reveal the molecular mechanism of sorbicillinoids production.

## Results

### Sorbicillinoids hyperproduction without affecting the cellulosic enzymes production in *T. reesei* mutant JNTR5

Although MFS permease *Tr69957* structurally resembles a maltose permease [28], it does not transport maltose, but cellobiose, xylose and mannose, and is reported to be involved in the degradation of cellulose [28]. These results suggest that *Tr69957* may be involved in the induction of cellulases. Deletion of gene *Tr69957* in *T. reesei* can decrease the expression of gene *cel7a*, *cel6a*, and *cel3a.* Based on these previous findings, we hoped to obtain cellulase hyperproduction strain by overexpression of gene *Tr69957* in RUT-C30.

**Fig. 1:**
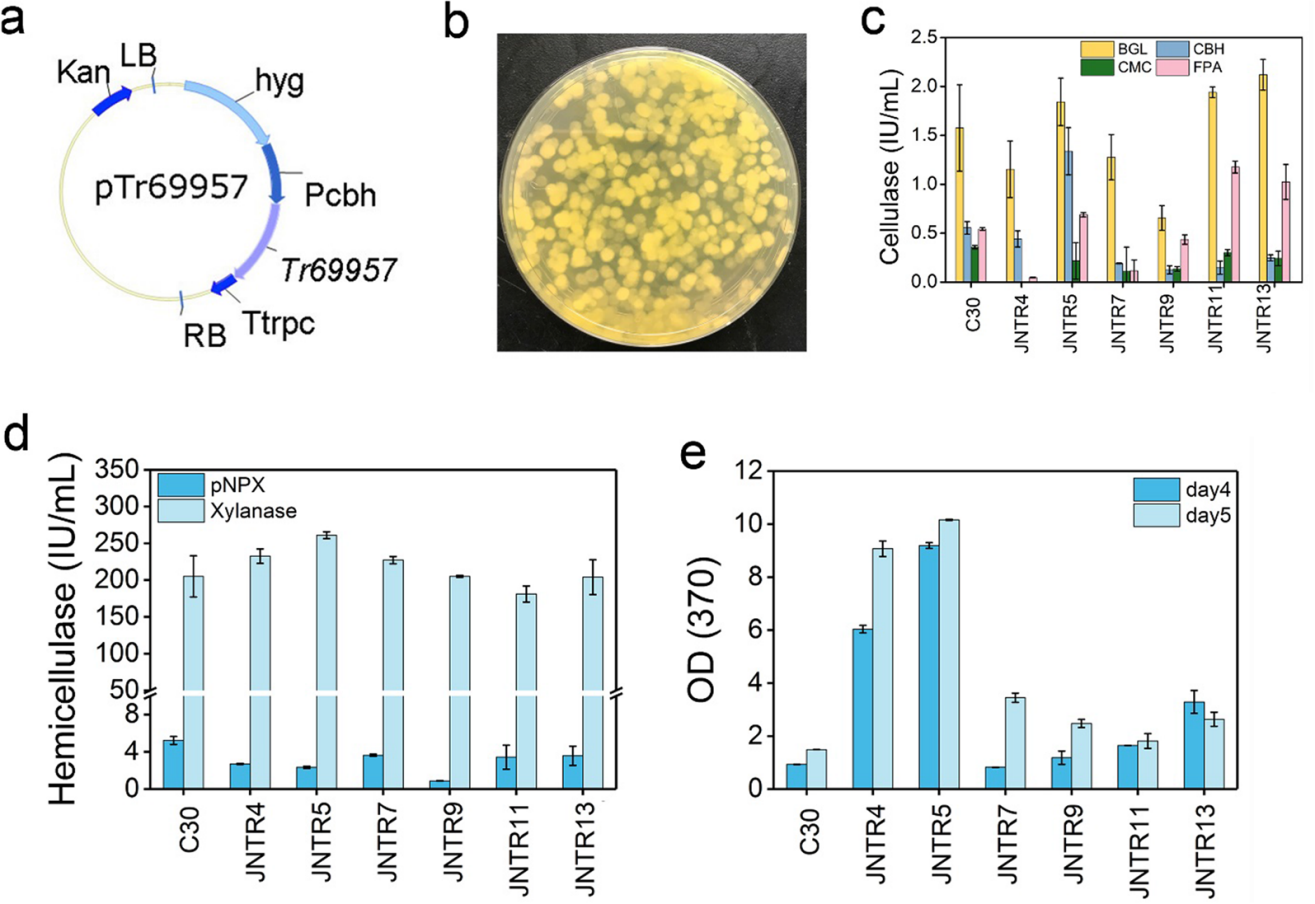
Construction of the plasmid-containing gene *Tr69957* and the transformation of strain *T. reesei* RUT-C30. **a** Schematic representation of the plasmid pTr69957. **b** The digital picture of the obtained *T. reesei* transformants by AMT method on PDA plate with 50 μg/mL Hyg B and 200 μM cefotaxime. **c**, **d** Cellulase and hemicellulase activities of *T. reesei* RUT-C30 and the transformants. The activities were determined on the 5th day. **e** The corresponding yellow pigments production of *T. reesei* RUT-C30 and the transformant strains

*Tr69957* was randomly inserted in the genome of *T. reesei* RUT-C30 by using the plasmid pTr69957 (Fig. 1a) to obtain the recombinant strains (Fig. 1b). Six transformants, JNTR4, JNTR5, JNTR7, JNTR9, JNTR11, and JNTR13 were finally selected. We detected the cellulase and hemicellulase production of the six transformants on cellulose. As shown in Fig. 1c, d, the pNPGase, pNPCase, CMCase, FPase, pNPXase and xylanase activities of all the six transformants on day 5 were in the range of 0.7–2.1, 0.1–1.3, 0–0.4, 0–1.2, 0.9–3.6, and 181.1–232.4 IU/mL, respectively. Unfortunately, the cellulase and hemicellulase activities were not enhanced in the transformants compared with that of RUT-C30, which are 1.6, 0.6, 0.4, 0.5, 5.2, and 205 IU/mL, respectively. However, during the growth of the transformants on PDA plates, we found that these recombinant strains exhibited much stronger yellow color on PDA plates than RUT-C30 (Additional file 1: Fig. S1), indicating far more yellow pigments were produced [13]. For quantitative comparison, the OD_370_ of the culture supernatants on day 4 and 5 was recorded on cellulose [24]. As shown in Fig. 1e, the sorbicillinoid production in strains JNTR4, JNTR5, JNTR7, JNTR9, JNTR11, and JNTR13 was 6.1, 6.8, 2.3, 1.7, 1.2 and 1.8-fold that of RUT-C30 on day 5.

All these results showed that overexpression of gene *Tr69957* increased the sorbicillinoid production without apparent influence on cellulase and hemicellulase production. The recombinant strain JNTR5 was selected for further study, for it displayed the highest yellow pigment production and comparable cellulase and hemicellulase production (Fig. 1). We detected the copy number of *Tr69957* in JNTR5 by qPCR. The results showed that the copy numbers of gene *Tr69957* inserted were 1 (Additional file 1: Fig. S2a). In addition, from the transcription result as shown in Additional file 1: Fig. S2b, we can see the expression of gene *Tr69957* in JNTR5 was much higher than that of RUT-C30, indicating the successful overexpression of *Tr69957*.

### Fluorescence characteristic of sorbicillinoids

**Fig. 2:**
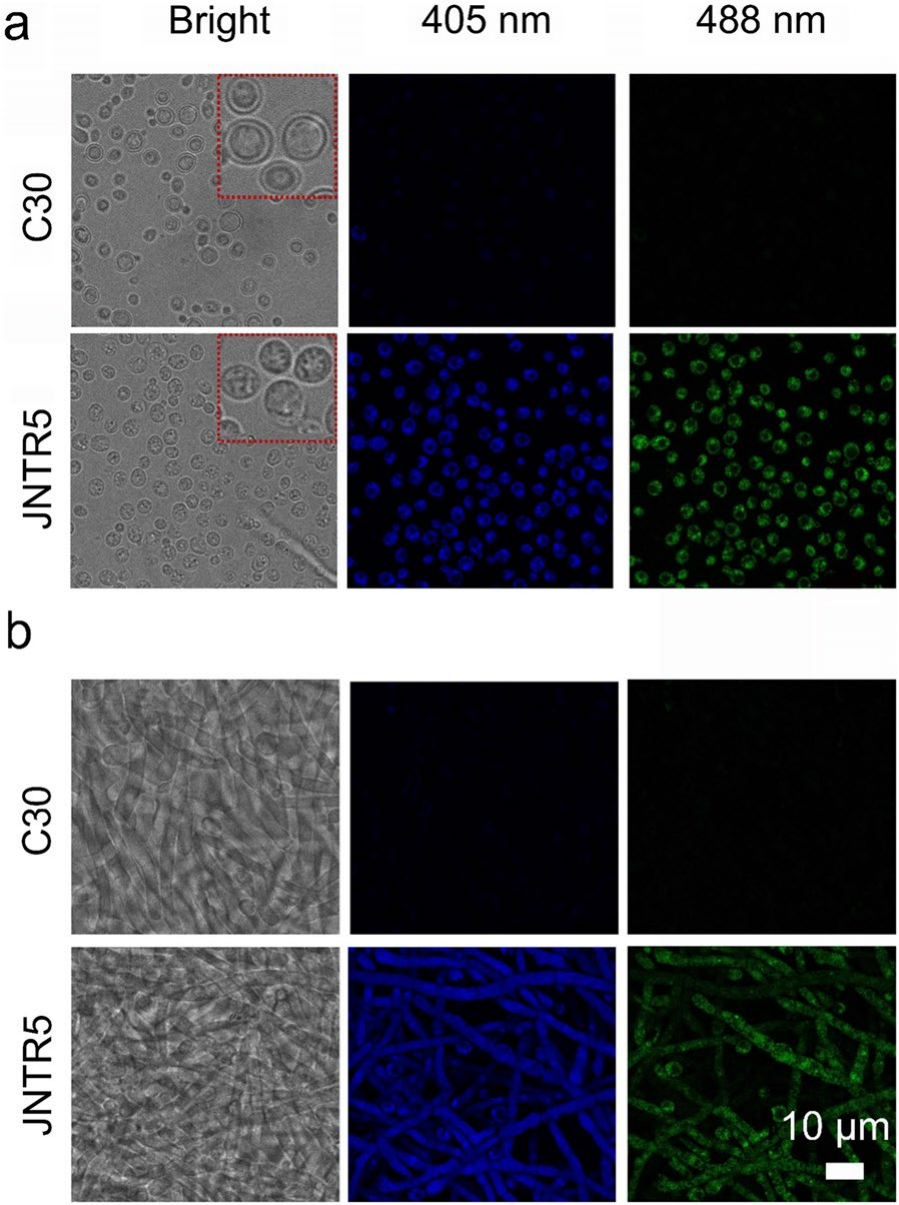
Confocal observation of yellow pigments production in strain RUT-C30 and JNTR5 at Ex/Em = 405/415–497 nm and 488/498–563 nm. **a** Confocal microscope images of spores of strain RUT-C30 and JNTR5. **b** Confocal microscope images of hyphae of strain RUT-C30 and JNTR5. Zoom factor = 2.5, frame averaging = 3. The area marked with the red square in A was the enlarged images for the spores of RUT-C30 and JNTR5, respectively. The samples were obtained by cultivating RUT-C30 and JNTR5 at 28 °C for 72 h with glucose as carbon source

When visualizing the aqueous solution of the yellow pigments under 302 nm UV light, we observed obvious emissive property with bright blue emission, and the emission showed concentration-caused emission quenching (Additional file 1: Fig. S3). Then, CLSM was used to observe the yellow pigment distribution in *T. reesei* RUT-C30 and JNTR5. The whole mycelium of JNTR5 exhibited strong blue and green fluorescence under 405 nm and 488 nm excitation, respectively (Fig. 2 and Additional file 1: Fig. S4). Interestingly, we also observed that the spores of JNTR5 displayed strong fluorescence, showing the yellow pigments were produced in the spores (Fig. 2a). For RUT-C30, no blue fluorescence was observed either in the mycelium or the spores (Fig. 2). Moreover, It was found that the cell wall of JNTR5 spores seemed to be compromised as shown in the enlarged images (Fig. 2a), which may benefit the secretion of cellulase/hemicellulase[29].

### Effects of carbon sources and light on the production of sorbicillinoids in JNTR5

**Fig. 3:**
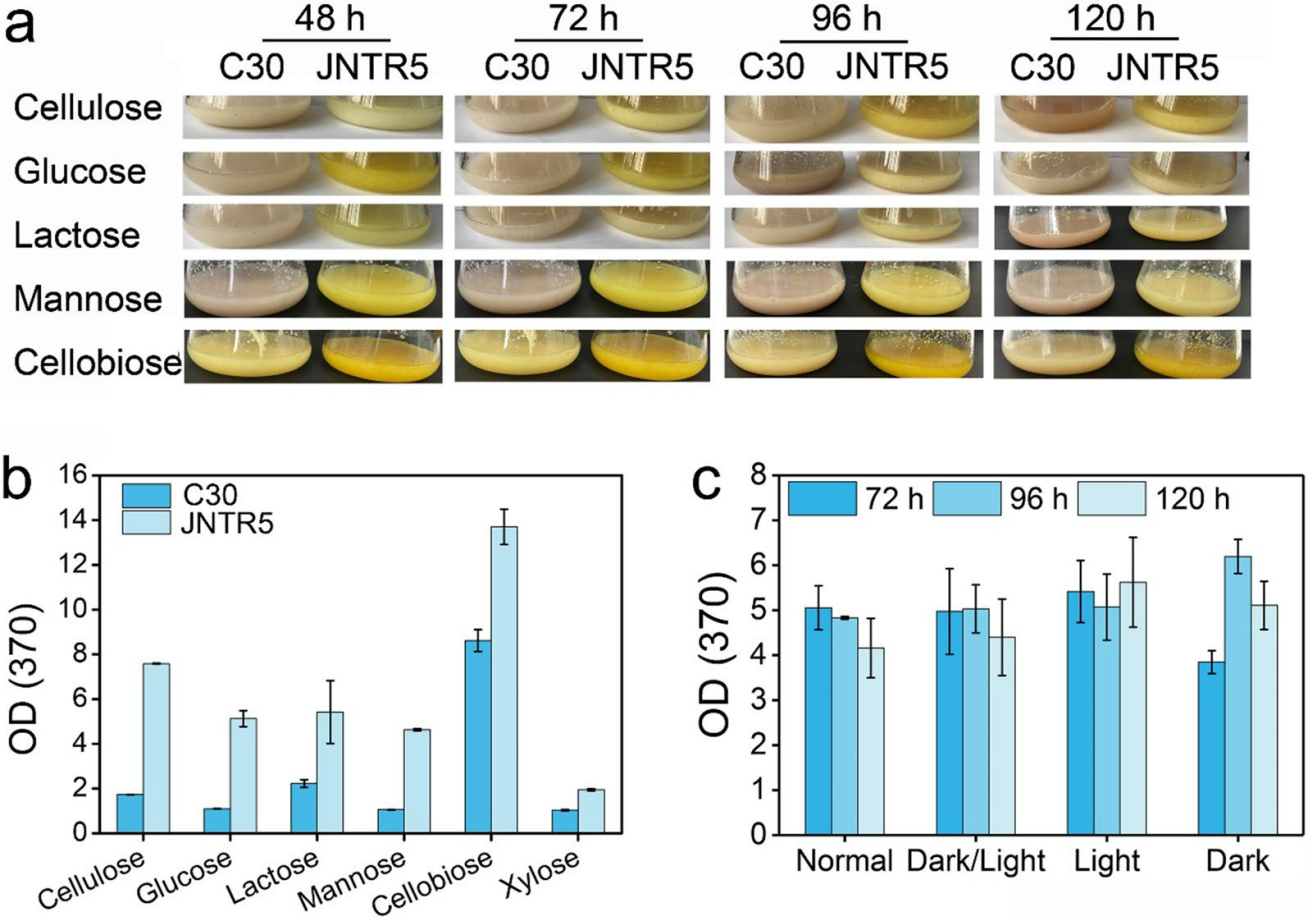
Influence of carbon sources and lights on the sorbicillinoids production. **a** The digital pictures of the culture supernatants of strain RUT-C30 and JNTR5 grown on different carbon sources, including cellulose, glucose, lactose, mannose, cellobiose, and xylose, respectively. **b**, **c** The sorbicillinoids production in *T. reesei* RUT-C30 and JNTR5 grown on TMM with different carbon sources on day 4 and day5

Carbon source is an important factor that influences the growth, cellulase production, and other secondary metabolite productions in fungi [30]. Sorbicillinoid production by *T. reesei* varies with different carbon sources [31, 32]. In order to evaluate the influence of carbon sources on the production of sorbicillinoids, we determined the sorbicillinoid production of RUT-C30 and JNTR5 grown on different carbon sources, including cellulose, glucose, lactose, mannose, cellobiose, and xylose (Fig. 3). Pictures of the cultures were also taken at different time courses ranging from 48 to 120 h (Fig. 3a). All transformants produced transparent yellow pigments compared with RUT-C30 during the time courses on different carbon sources. Interestingly, we found that cellobiose can induce most yellow pigments production not only for JNTR5 but also for RUT-C30, which may be due to the activation of some silent genes in *T. reesei* by changing the cultivation condition [33]. We subsequently relatively quantified the sorbicillinoids of the culture supernatants on day 4 by measuring the value of OD_370_ (Fig. 3b). The OD_370_ of the culture supernatant of strain JNTR5 increased as time extended from day 4 to day 5. On day 5, the OD_370_ was 7.6, 5.1, 5.4, 4.6, 13.7, and 1.9 for cellulose, glucose, lactose, mannose, cellobiose and xylose, respectively, which was 4.5, 4.6, 2.5, 1.6, 2.7 and 1.9-fold that of RUT-C30 (Fig. 3c). Apparently, the production of sorbicillinoids by strain JNTR5 on all tested carbon sources increased significantly. Sorbicillinoid production with cellobiose as the carbon source was the highest, followed by cellulose, lactose, glucose, mannose, and xylose in descending order.

To determine the influence of light on its sorbicillinoid production, strain JNTR5 was grown on TMM + 2% glucose under normal light in the lab, constant light, constant darkness, or cycles of 12 h light/12 h darkness for 120 h. The absorbance of the supernatants at 370 nm was not changed under different conditions, which is similar to that of strain ZC121 [24]. The highest value of OD_370_ = 6.2 was observed for JNTR5 grown under constant darkness for 96 h, whereas the values under other conditions and time courses were comparable (Fig. 3d). Cellulase and hemicellulase production was also not influenced on different light conditions (Additional file 1: Fig. S5). In short, the hyperproduction of sorbicillinoids in strain JNTR5 is independent of both carbon sources and light.

### Characterizations of *T. reesei* JNTR5

**Fig. 4:**
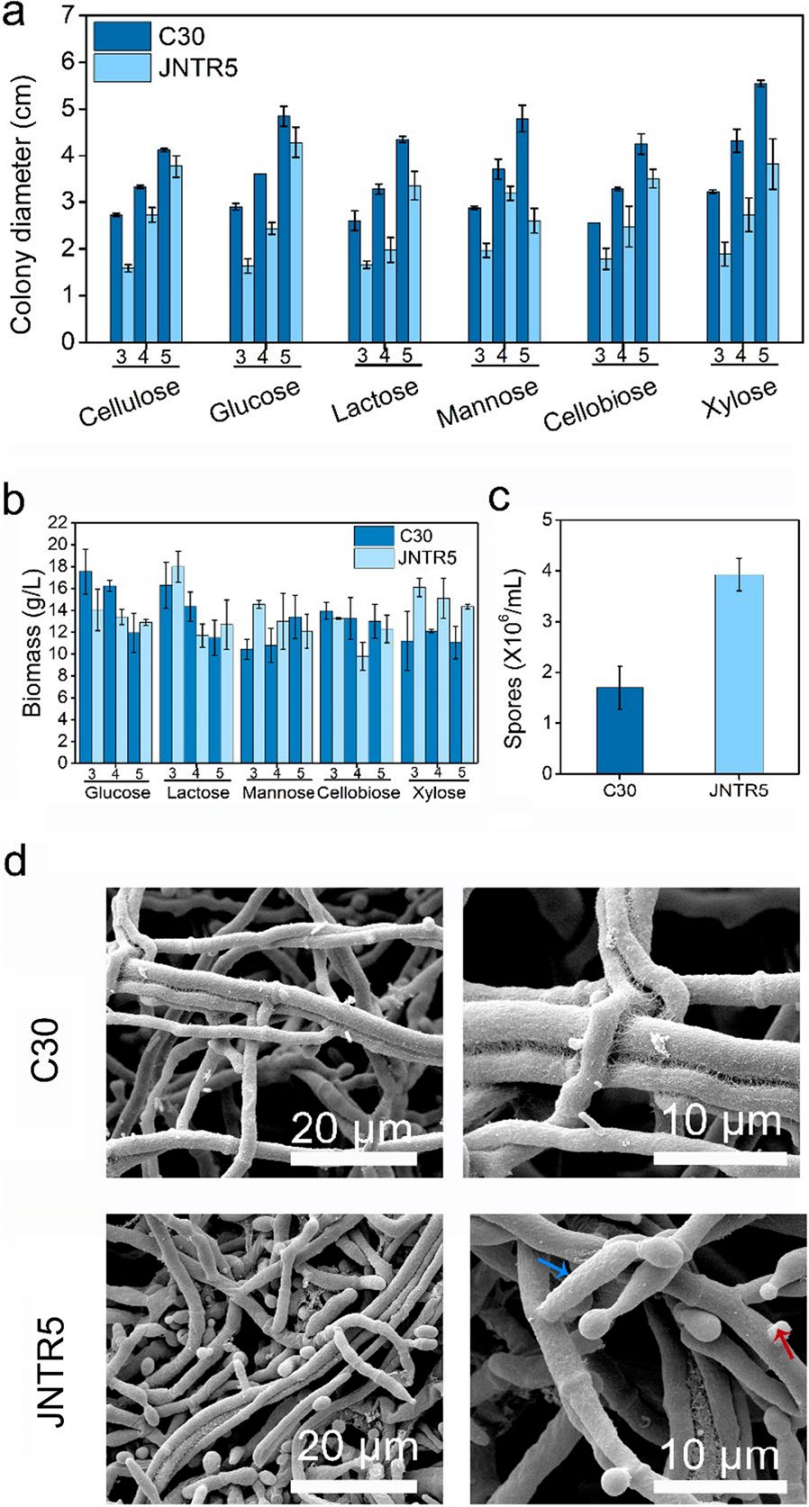
Characterizations of strain RUT-C30 and JNTR5. **a** The conidial growth of strain RUT‑C30 and JNTR5 on different carbon sources by measuring the colony diameters on day 3, day 5, and day 7. **b** Biomass accumulation of strain RUT-C30 and JNTR5 on different time courses. **c** Spore production of strain RUT‑C30 and JNTR5. **d** The morphological structures of *T. reesei* RUT-C30 and JNTR5 observed by SEM. The blue and red arrowheads indicate the branch and conidia heads. Error bars indicate SDs from three independently cultured replicates

Growth of strain RUT-C30 and JNTR5 was examined by measuring colony diameters and biomass accumulation on TMM plates containing different carbon sources, including cellulose, glucose, lactose, mannose, cellobiose, and xylose. The colony diameters of JNTR5 were less than that of RUT-C30 on different carbon sources, indicating that the overexpression of gene *Tr69957* affected the growth of *T. reesei* (Fig. 4a). Biomass accumulation was studied by measuring the dry weight of mycelium of strain RUT-C30 and JNTR5 on soluble carbon sources. Little fluctuation in biomass accumulation between RUT-C30 and JNTR5 was observed in the presence of all detected carbon sources at different time courses (Fig. 4b). Furthermore, secondary metabolism is commonly associated with the sporulation process in fungi [34]. Thus we investigated the spore production on PDA plates. The spore amount of strain JNTR5 was 3.9×10^6^/mL, which was a little higher than that of RUT-C30 (1.6×10^6^/mL) (Fig. 4c), exhibiting that the overexpression of gene *Tr69957* results in the noticeable increase of sporulation in *T. reesei* JNTR5. Consistent result was also observed from the confocal images of JNTR5 and RUT-C30. The positive link between pigment production and spore production in fungi was also found in other fungi, like *Chlorociboria aeruginascens* [35].

The SEM observation was further carried out to observe the cell morphology of *T. reesei* RUT-C30 and JNTR5. The surface of JNTR5 was smooth and partially covered with fibrous material, different from that of RUT-C30 with more fibrous appearance in the surface of the hyphae. It was also confirmed from SEM images that the hyphae of strain JNTR5 were highly branched and elongated (Fig. 4d), resulting in more conidia heads containing vesicles, conidiophores, and sterigma. Overall, with the overexpression of gene *Tr69957*, the biomass of strain JNTR5 on the soluble carbon sources was not affected significantly, but the conidial growth was negatively affected, the spore formation increased and the morphology changed.

### Transcription patterns of strain JNTR5

**Fig. 5:**
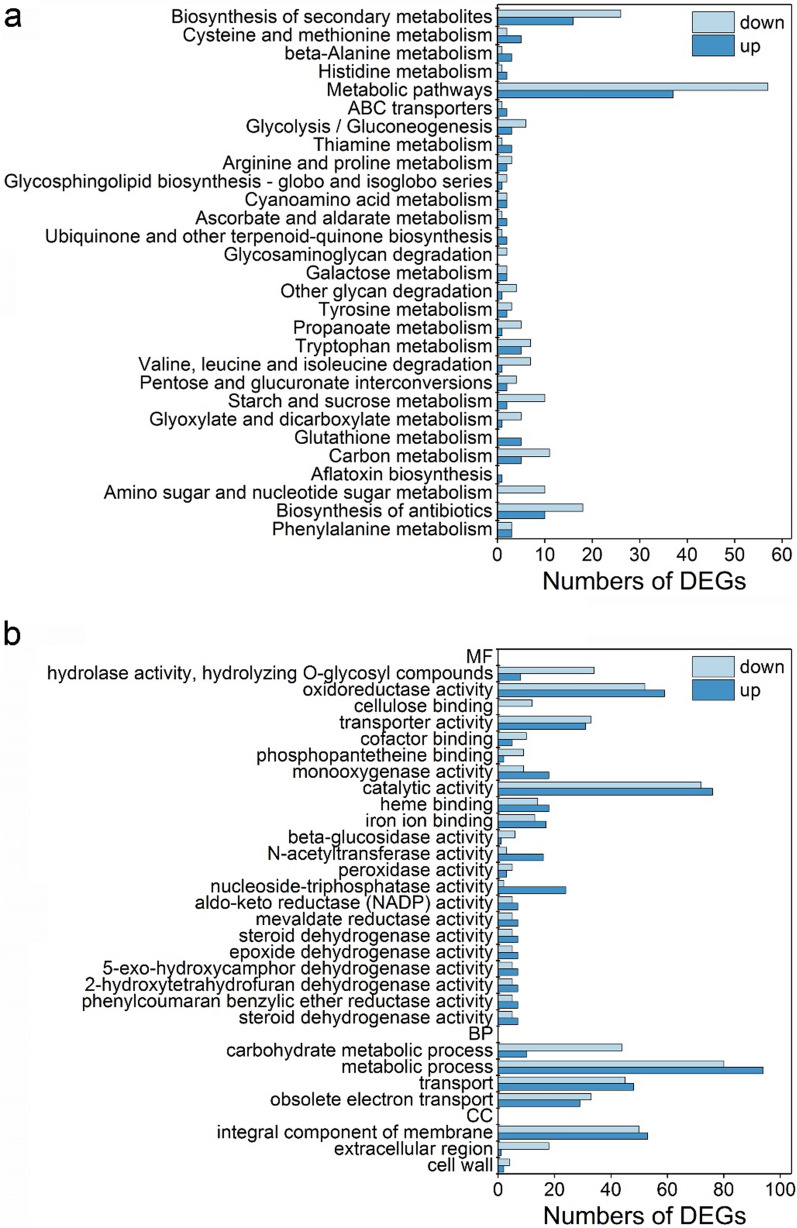
Kyoto Encyclopedia of Genes and Genomes (KEGG) pathway (**a**) and Gene Ontology (GO) analysis (**b**) of DEGs in strain JNTR5 and RUT‑C30 on cellulose. MF (molecular function), BP (biological process), and CC (cellular component) are the GO terms

In order to understand the influence of overexpression of gene Tr69957 on cellulase or sorbicillinoid production under the transcriptional level, RNA-seq was performed for strain RUT-C30 and JNTR5 grown on cellulose. The total reads were mapped to the *T. reesei* RUT-C30 reference genome resulting in 71.66–94.17% coverage of the reference genome. We obtained 1477 differentially expressed genes (DEGs) with 705 upregulated genes and 772 downregulated genes by comparing the genome of RUT-C30 and JNTR5 under cellulose growth condition. In order to categorize the differentially expressed genes into functional pathways, we performed the KEGG enrichment analysis. The top 30 pathway results of RUT-C30 and JNTR5 on cellulose were selected and presented in Fig. 5a. The result shows that most DEGs are enriched in the “Biosynthesis of secondary metabolites” and “Metabolic pathways”. This result is consistent with findings on the increasing of sorbicillinoids production in strain JNTR5 compared with RUT-C30.

These DEGs for RUT-C30 and JNTR5 were further analyzed using GO-TermFinder to find the detailed information of the DEGs, identifying Gene Ontology (GO) terms (Fig. 5b). For the enriched molecular function, the enriched “cellulose binding”, “hydrolase activity” and “cellulase activity” were observed on cellulose, of which all genes were downregulated, indicating that overexpression of gene *Tr69957* inhibited the expression of cellulase-related genes. The “carbohydrate metabolic process” belonged to the enriched biological process and most of them were downregulated. For cellular component, 103, 19, and 6 DEGs were enriched in “integral component of membrane”, “extracellular region”, and “cell wall”, which is reasonable for that both cellulases and sorbicillinoids are secreted into the culture media through the fungal cells. Such enriched DEGs are also consistent with the results shown in Fig. 3a that the spore cell wall and cell surface of JNTR5 seems to be different from that of RUT-C30.

### Most DEGs involved in sorbicillinoid biosynthesis were upregulated in *T. reesei* JNTR5 on cellulose

Based on the results of the transcriptional analysis, the cellulase and hemicellulase encoding genes, such as gene *cel6a* encoding cellobiohydrolase, gene *egl1*, *egl2*, *egl3*, *egl4*, and *egl5* encoding endoglucanases, β-glucosidases encoding genes *cel3a*, *cel3d*, *cel7a*, gene *bxl1* encoding β-xylosidase, *xyn1*, *xyn2* and *xyn3* encoding xylanases were all downregulated significantly in strain JNTR5 (Table 1). Besides, genes *swo1*, *cip1* and *cip2* essential for improving the cellulose degradation were also downregulated in JNTR5. However, the expressions of the cellulase transcription activators XYR1, ACE1, ACE2, ACE3, CRT1, HAP3 and HAP5 were not changed significantly, while the mRNA level of *cel3e* was upregulated.

**Table 1:** The main DEGs related to yellow pigments, cellulase, and hemicellulase on cellulose

Gene name	Gene ID	logFC	*p* Value
Sorbicillinoids-related genes			
* sor5*	M419DRAFT_67737	–	–
* sor1*	M419DRAFT_93844	5.03	1.86E-30
* sor2*	M419DRAFT_93847	6.68	8.35E-43
* sor3*	M419DRAFT_139626	13.14	3.65E-76
* sor6* (MFS)	M419DRAFT_121436	5.63	1.57E-34
* ypr2*	M419DRAFT_31634	4.20	4.08E-30
* sor4*	M419DRAFT_4579	7.34	1.62E-68
* ypr1*	M419DRAFT_93861	0.99	0.000152
Cellulase-related genes			
* cel6a*	M419DRAFT_122470	− 2.76	8.74E-16
* egl1*	M419DRAFT_5304	− 3.35	5.23E-22
* egl2*	M419DRAFT_124931	− 2.28	4.69E-12
* egl3*	M419DRAFT_72489	− 3.01	6.72E-19
* egl4*	M419DRAFT_139633	− 1.36	8.90E-05
* egl5*	M419DRAFT_25940	− 3.07	2.92E-16
* cel3a*	M419DRAFT_136547	− 3.76	2.61E-27
* cel3d*	M419DRAFT_122639	− 1.15	0.000135229
* cel3e*	M419DRAFT_74305	1.07	0.000412925
* cel61b*	M419DRAFT_122518	− 2.92	1.43E-12
* cel1a*	M419DRAFT_127115	0.59	0.050495
* cel1b*	M419DRAFT_77989	0.23	0.430264
* cel7a*	M419DRAFT_125125	− 2.6	5.32E-12
* xyr1*	M419DRAFT_98788	− 0.7	0.008064
* crt1*	M419DRAFT_109243	0.71	0.01742
* swo1*	M419DRAFT_104220	− 2.32	3.78E-12
* cip1*	M419DRAFT_121449	− 2.04	3.17E-08
* cip2*	M419DRAFT_125575	− 2.87	9.60E-10
Hemicellulase-related genes			
* xyn1*	M419DRAFT_38418	− 1.92	1.05E-08
* xyn2*	M419DRAFT_124931	− 2.28	4.69E-12
* xyn3*	M419DRAFT_23616	− 3.83	1.39E-25
Acetylxylan esterase axe1	M419DRAFT_139631	− 1.40	1.01E-05
Acetylxylan esterase	M419DRAFT_88887	− 3.18	3.98E-15
α-N-Arabinofuranosidase abf1	M419DRAFT_102517	− 1.47	0.000124
β-Xylosidase	M419DRAFT_77521	− 1.63	4.31E-05
α-L-Arabinofuranosidase abf2	M419DRAFT_118070	− 2.81	8.24E-14
Mannan endo-1,4- β-mannosidase	M419DRAFT_122377	− 2.76	9.31E-16
Xyloglucanase	M419DRAFT_111943	− 2.67	4.12E-15
Glucuronoxylanase xynC	M419DRAFT_93498	− 3.39	2.17E-16
α-Glucuronidase	M419DRAFT_90302	− 1.56	4.28E-07
TR69957	M419DRAFT_91594	0.997483	0.000674

Genes associated with the biosynthesis of sorbicillinoids were clustered in the genome of *T. reesei,* which were named “sorbicillinoids gene cluster” [24, 27]. This sorbicillinoids gene cluster contained 8 genes, of which two genes (*ypr1* and *ypr2*) are two Gal4-like transcription factors (TFs). Other genes include the highly reducing/non-reducing PKS (*sor1*/*sor2*), *sor3* encoding a FAD-dependent monooxygenase, an FAD-binding dehydrogenase *sor4*, a short-chain dehydrogenase/reductase encoding gene *sor 5*, and a MFS transporter *sor6* [24, 25]. Different from the (hemi)cellulase-encoding genes, all these genes were upregulated significantly in strain JNTR5, which was consistent with the excellent sorbicillinoid production capacity for JNTR5 as shown in Fig. 3.

In addition to YPR1, we also detected the expression of XPP1 acting as an activator for primary metabolism and repressor for the second metabolism on cellulose, lactose and glucose. On cellulose, the expression of XPP1 between JNTR5 and RUT-C30 exhibits no differences, indicating that the regulation of Tr69957 on pigment secretion and production was uncorrelated to XPP1. Meanwhile, Sor 5, which was not found in most other fungi, nearly had no expression in both RUT-C30 and JNTR5, which was different from ZC121 in which sor5 was activated [24].

**Fig. 6:**
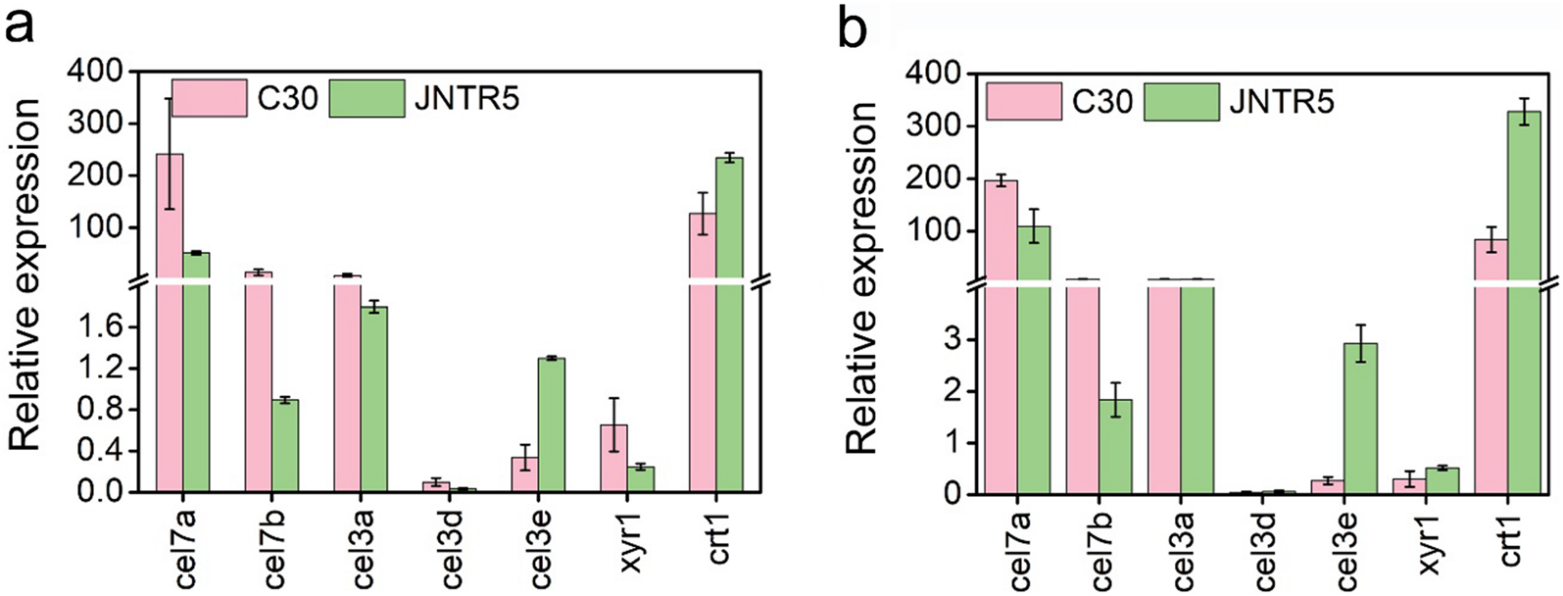
qRT-PCR analysis of the expression of genes relevant to cellulase and hemicellulase production in *T. reesei* RUT-C30 and JNTR5 grown on 2% cellulose on day 3 (**a**) and day 4 (**b**)

Moreover, we analyzed the transcription of genes related to cellulase production, including cellulase-encoding genes (*cel7a*, *cel7b*, *cel3a*, *cel3d*, and *cel3e*), cellulase transcription activator *xyr1*, and sugar transporter *crt1* (Fig. 6). All cellulase-encoding genes were downregulated in JNTR5 on day 3 except for *cel3e* (Fig. 6a). Gene *xyr1* was downregulated in JNTR5, but the expression of *crt1* was upregulated. The results coincide with that of RNA-seq result (Table 1). On day 4, the expression of cellulase-encoding genes *cel3a*, *cel3d* in JNTR5 increased to be comparable with that in RUT-C30, and *cel3e* was significantly upregulated (Fig. 6b), and *xyr1* and *crt1* were upregulated in JNTR5. However, the expression of *cel 7a* and *cel7b* was still reduced in JNTR5.

#### Cell wall integrity of JNTR5

**Fig. 7:**
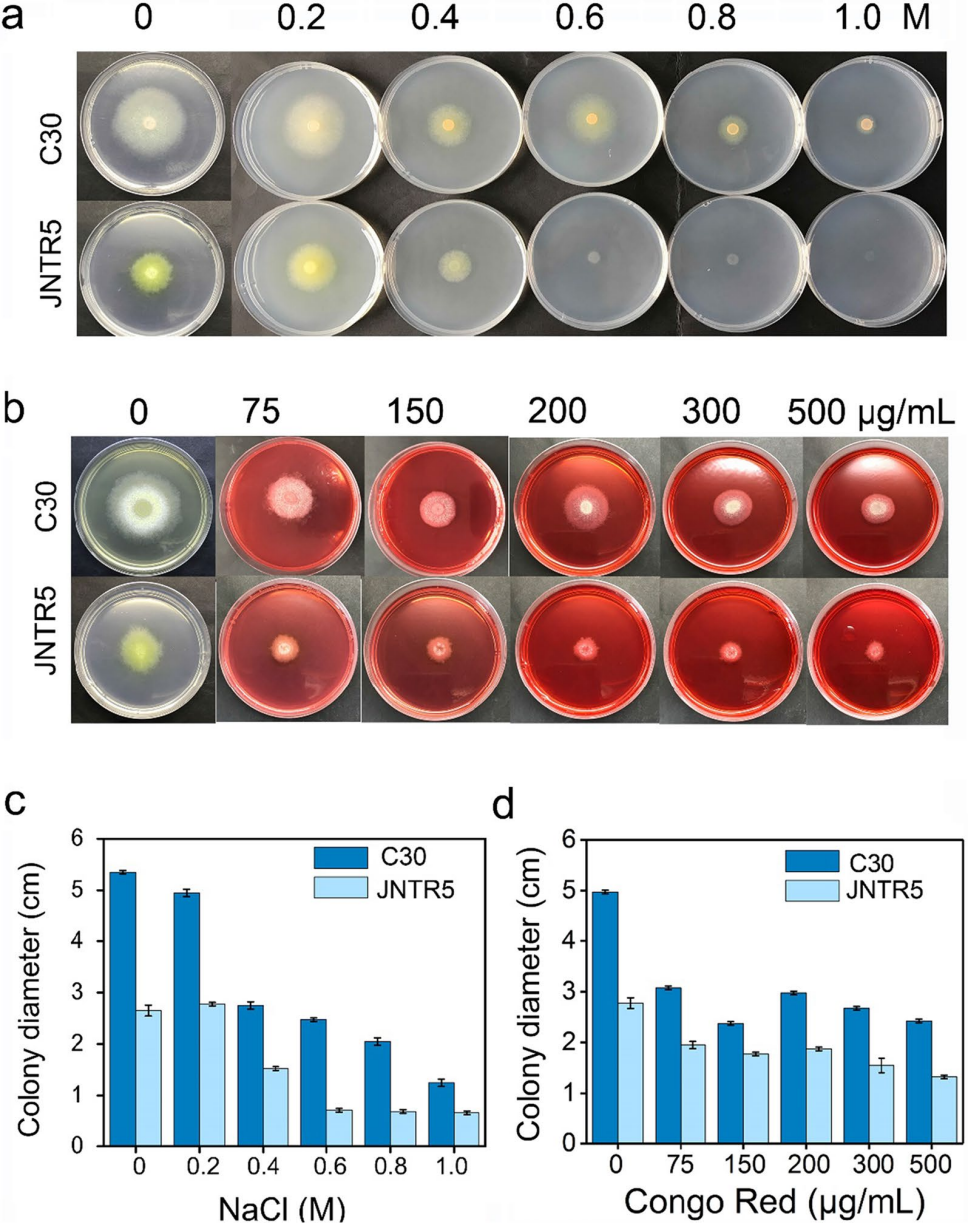
The susceptibility of RUT-C30 and JNTR5 to NaCl or CR. **a**, **b** Images of *T. reesei* RUT‑C30 and JNTR5 grown on PDA plates containing different content of NaCl or CR for 72 h. **c**, **d** Colony diameter statistics of *T. reesei* RUT‑C30 and JNTR5 grown on PDA plates containing different content of NaCl or CR for 72 h

As shown in Fig. 7, with the increasing concentrations of NaCl on PDA plates, the growth of JNTR5 mutant strain was inhibited compared with the parental strain RUT-C30 (Fig. 7a, c), suggesting the higher sensitivity of JNTR5 to NaCl than RUT-C30. This result further suggested that the cell wall of JNTR5 might be compromised, and therefore no longer supported the normal growth of JNTR5 on plates containing higher concentrations of NaCl. Furthermore, RUT-C30 could tolerate at least 500 μg/mL CR, while the sensitivity of JNTR5 to CR increased when 200 μg/mL CR was used, indicating that the cell wall of JNTR5 may be damaged (Fig. 7b, d). This also gives an explanation for the comparable cellulase/hemicellulase production between JNTR5 and RUT-C30 even with the down-regulation of the related encoding genes in JNTR5.

## Discussion

Sorbicillinoids are one class of the microbial secondary metabolites with diverse valuable bioactivities like anticancer, antimicrobial, antivirus, and radical-scavenging activities [7]. Extensive studies on finding similar new compounds, structure illustration, and chemical synthesis are continuously proceeding. However, scientists ignored the natural producing source of sorbicillinoids, and less studies were performed on the large-scale microbial production and the regulatory mechanism. *T. reesei* is recognized as a workhorse for producing cellulase and studying the production mechanism [29]. Meanwhile, they can also produce sorbicillinoids during growth, which can be used for constructing genetic engineering strains for producing sorbicillinoids by utilizing biomass and studying the production mechanism as reported in our previous study [24].

Some microbial pigments are known to have fluorescent properties, which can be used to directly track the localization, secretion pathway of the pigments in living organism, and will lead to a better facilitation and deeper understanding of the molecular mechanism behind the pigments production [36]. It has been reported that the yellow pigments produced in the mycelium of *T. reesei* ZC121 exhibited blue fluorescence under 405 nm excitation [24]. The fluorescence properties of such yellow pigment may expand its application in various fields, like bioimaging, biosensing, and chemo-sensing. Nevertheless, the exact distribution in organelle and the underlining secretion pathway of sorbicillinoids have not been clear.

Moreover, Schmidt et al. have reported that fungal mycelium is able to respond to light, leading to the production of mature conidia [35]. Light signaling cascades are also involved in the regulation of growth, development and enzyme production in *T. reesei* [37]. Some regulators have been shown to be involved in the regulation of secondary metabolites affecting by light, like SUB1 responsible for negative regulation of trichodimerol production specifically in darkness, but not in light [38]. YPR2 is a regulator balancing secondary metabolism with carbon metabolism in darkness of *T. reesei* [39].

In the present study, the recombinant strain JNTR5 with sorbicillinoids hyperproduction ability was constructed. Different from our previous mutant strain ZC121 that exhibited enhanced sorbicillinoids production with decreased cellulase production [24], JNTR5 produced enhanced sorbicillinoids without significantly affecting cellulase/hemicellulase production. When the sorbicillinoids encoding genes were activated, the cellulase/hemicellulase encoding genes were inhibited at the initial culture stages. When the strains were cultured for 4 days, the BGL encoding genes were upregulated, but the expression of *cel7a* and *cel7b* was still decreased in JNTR5. Nevertheless, the expression of their isoenzymes remained unchanged from the transcription result (Additional file 2: Table S2), which may contribute to the almost unchanged cellulase production. Based on this interesting finding, strain JNTR5 can serve as a new platform for studying the production mechanism of secondary metabolites and the conversion mechanism between different secondary metabolites. JNTR5 also expands the model industrial microorganisms for desired compound production by single microorganism to degrade cellulosic plant biomass.

When glucose was used as carbon source for the production of sorbicillinoids, OD_370_ = 5.1 for the supernatant of JNTR5 is lower than that of *T. reesei* strain ZC121 and the *xpp1* gene knockout strain with OD_370_ = 17.3 and 13, respectively (25, 26). However, when grown on cellulose, strain JNTR5 produced more sorbicillinoids of OD_370_ = 7.6, which was higher than the cultures with the deletion of gene *xpp1* with OD_370_ ≈ 0.32 [25]. Interestingly, when cellobiose was used as carbon source, both RUT-C30 and JNTR5 can produce amounts of sorbicillinoids with the OD_370_ = 8.6 and 13.7, respectively. Such enhancement in both strains indicate that cellobiose may be a key factor for activating some silent genes responsible for secondary metabolites, needing to be investigated in future. Cellobiose is recognized as a natural inducer for cellulase production [40]. A low-concentration of cellobiose can effectively induce the expression of cellulase in *T. reesei* [41]. Cellobiose is also recognized as the main inducer of CAZy genes in a *D. squalens dikaryon* [42]. In *Neurospora crassa*, the deletion of β-glucosidase gene allows cellobiose to induce the cellulase gene to the same level as induced by cellulose [43]. The highest sorbicillinoid productivity of JNTR5 was found when using cellobiose as the carbon source, which might be due to that Tr69957 can transport cellobiose [28] to increase the cellobiose access and utilization. Similar results have been reported in the literature. For example, homologous expression of a sugar transporter gene (*Pshxt1*) in the white rot fungus *Phanerochaete sordida* YK-624 improved aerobic ethanol production [44]. In another study, overexpression of a glucose transporter GLT-1 or the cellodextrin transport system (CDT-1/CDT-2) from *N. crassa* increased ethanol production in *Myceliophthora thermophila* by 131% on glucose or by 200% on cellobiose, respectively [45]. The sorbicillinoids production in fungi has been reported to be generally impacted by culture conditions, such as carbon source [13], and light [46]. By contrast, the recombinant JNTR5 displayed hyperproduction of sorbicillinoids regardless of the carbon source or the exposure to light. This constitutive hyperproduction will benefit the future industrial application of strain JNTR5 for sorbicillinoids production with great flexibility and easy fermentation operation.

Secondary metabolism is commonly associated with sporulation processes in fungi [34, 47]. In our previous study, we have reported that in strain ZC121, off-target mutagenesis leads to noticeable reduction of sporulation [24]. However, JNTR5 produced more spores than RUT-C30. This positive link between secondary metabolites production and sporulation in fungi is common [47]. Overall, with overexpression of gene *Tr69957*, the radical growth rate and biomass accumulation of JNTR5 are not affected significantly, but its morphology changes with marked enhanced sporulation. The morphological changes caused by the membrane protein overexpression have been reported in previous reports. For example, It has been reported that the type I integral plasma membrane protein Axl2 (axial budding pattern protein 2) deleted *Acremonium chrysogenum* strain exhibited swollen and highly septated hyphae at 72 h and its arthrospore formation was accelerated at 96 h in liquid culture [48].

The fungal cell wall is the first layer that interacts with the environment and the enhanced production of secondary metabolites can be also related to the damage of cell wall integrity [49]. Consequently, cell wall stress is potentially responsible for the induction of a series of stress-responsive factors, including the production of secondary metabolites [50]. It has been reported that Tmk2 maintains cell wall integrity, and negatively impacts cellulase secretion because of the intact cell wall [28]. Similar results have been also reported by Hongting Tang [51] that the enhancement of protein secretion was accomplished by damaging cell walls. In JNTR5, the comparable cellulase and hemicellulase production under the decreased expression of related genes also resulted from the damage of cell wall integrity. Thus it can be suggested that by engineering the cell wall structure or hampering cell wall integrity, the secretion of secondary metabolites becomes easy because the metabolites can easily penetrate cell wall, thereby leading to the enhancement of secreted metabolites.

## Conclusions

We constructed a recombinant *T. reesei* strain JNTR5 from RUT-C30 by random insertion of gene Tr69957 with AMT method. Strain JNTR5 exhibited significant enhanced yellow pigments production on either cellulose or other soluble carbon sources when compared with RUT-C30 with only a little reduction in cellulase and hemicellulase production. The hyperproduction of sorbicillinoids was not influenced by light. JNTR5 produced more spores than RUT-C30 and exhibited enhanced biomass accumulation and growth rate. The sorbicillinoids were distributed in both mycelium and spores of strain JNTR5 with blue and green fluorescence when excited with 405 nm and 488 nm laser. Consistently, the transcriptional level of genes involved in sorbicillinoid production was upregulated, and the genes involved in most cellulase and hemicellulase were observed downregulated at the transcriptional level in JNTR5.

## Materials and methods

### Strains, plasmids, and culture media

*T. reesei* parent strain RUT-C30 (CICC 13,052) was purchased from China Center for Industrial Culture Collection (CCICC). *Escherichia coli* DH5α (Vazyme, China) chemically competent cells were used as cloning vector. *Agrobacterium tumefaciens* AGL1 chemically competent cells (Weidi Bio, China) were used as a T-DNA donor for transformation [52]. Plasmid pDht/sk was friendly provided by Professor Zhihua Zhou (CAS Key Laboratory of Synthetic Biology, Shanghai) and was used to construct the recombinant strains. Hygromycin B (Hyg B) with a final concentration of 50 μg/mL was used as the selection marker.

*T. reesei* strains were grown on potato dextrose agar (PDA) plates with or without Hyg B at 28 °C for 7 days. Spores were harvested with sterilized 0.02% Tween 80 and stored in 33% glycerol at − 80 °C. Bacteria including *E. coli* DH5α and *A. tumefaciens* AGL-1 were, respectively, cultured overnight in Luria–Bertani (LB) medium at 37 °C and 28 °C, 200 rpm [26].

### Construction of *T. reesei* mutant strains

Total RNA and cDNA was obtained according to our previous methods [28]. Gene *Tr69957* was cloned with the cDNA of RUT-C30 as template. The primers used for gene amplification are presented in Additional file 1: Table S1. The resulting gene was treated with *Xba* I and ligated with *Xba* I-treated plasmid pDht/sk according to the instruction of the ClonExpress One Step Cloning Kit (Vazyme, China), obtaining the plasmid pTr69957 (Fig. 1A). The AMT method was used for transformation [50]. The transformants were selected on PDA plates with Hyg B (50 μg/mL) and cefotaxime (200 μM) [50]. Six *T. reesei* transformants JNTR4, JNTR5, JNTR7, JNTR9, JNTR11, and JNTR13 were successively obtained after additional five generations selection.

### Fungal cultivation

Spores (10^6^/mL) of RUT-C30 and JNTR5 were inoculated into 10 mL of Sabouraud dextrose broth (SDB) with or without Hyg B at 28 °C, 180 rpm for 48 h. Then 5 mL of the cell cultures were transferred into TMM media (50 mL) containing 2% (w/v) cellulose, glucose, lactose, mannose, cellobiose, and xylose with or without Hyg B and cultured under the same conditions [27]. The samples were centrifuged at 8000 rpm for 30 min at 4 °C and the culture supernatants were used for enzyme and sorbicillinoids analysis [24]. The mycelium was used for RNA-seq and biomass determination by calculating the dry weight of mycelium after drying by incubation at 70 °C. The pictures of cultures were also taken from 5 days cultivation. Cellulase/hemicellulase activities were determined according to the methods in our previous studies [26, 53], and the absorbance at 370 nm was detected to determine the amount of sorbicillinoids.

To compare the growth rates of RUT-C30 and JNTR5, strains were pre-grown on PDA plates with or without Hyg B at 28 °C for 5 days. An equal volume of the spore solutions (20 μL) were inoculated onto the center of TMM plates with 2% (w/v) different carbon sources (cellulose, glucose, lactose, mannose, cellobiose, and xylose), PDA plates (3 replicates), and cultured under the same conditions as mentioned above. Images of the plates were taken and the colony diameters were measured.

### Microscopy observation

RUT-C30 and JNTR5 were cultivated in TMM with 2% glucose as carbon source for 48 h and 72 h. The mycelia of above strains were immersed into a little drop of sterilized 30% glycerol placing on a glass slide, covered with a cover glass. Then, the slide was observed with an inverted confocal laser scanning microscope (CLSM) SP8 (Leica, Germany) with an oil-immersion objective lens (100 × 1.4 NA).

Cold field emission scanning electron microscope (FESEM) images of the fungi were obtained using a Hitachi SU8220 FESEM (Hitachi Co., Japan). Mycelium and spores of RUT-C30 and JNTR5 cultured on PDA plates for 4 days were scraped off into a centrifuge tube and 5% glutaraldehyde solution in 0.1 M phosphate buffer (pH 7.2) was used to fix cells for 4 h to preserve its original shape. Then, 1% osmic acid was used for another fixing for 2 h. After being dehydrated with ethanol solutions in 30, 50, 70, 80, 90, 95, and 100% (v/v), the samples were dropped on a silicon slide and air dried for FESEM examination.

### Sporulation assay

RUT-C30 and JNTR5 were cultivated on PDA plates at 28 °C until the mycelium was mature, and then collected with 4 mL of sterilized Tween 80 (0.02% v/v%). About 30 μL of spores were inoculated onto the center of new PDA plates with or without Hyg B and incubated at 28 °C for 5 days. The hemocytometer and light microscope were used to count the spores. Three biological replicates were carried out for each strain.

### Transcription analysis

TRIzol Reagent (Invitrogen, USA) and RNeasy Mini Kit (Qiagen, Germany) were used to extract the total RNA of RUT-C30 and JNTR5. After quantification of the obtained total RNA by NanoDrop (Thermo Fisher Scientific, USA), Agilent 2100 Bioanalyzer (Agilent Technologies, USA), and 1% agarose gel, 1 μg of total RNA (RIN > 7) was used for library preparation. Then, the library construction and sequences were carried out and analyzed with two duplicates by GENEWIZ (Su Zhou). The obtained raw data were submitted to the NCBI SRA database (BioProject accession number: PRJNA684017).

### Sensitivity to Congo red (CR) and NaCl

The preparation of spores was the same as the method mentioned above. About 20 μL of spore suspensions were inoculated on PDA plates with or without Hyg B, containing 0, 75, 150, 200, 200, 300, and 500 μg/mL CR or 0, 0.2, 0.4, 0.6, 0.8, 1.0 M NaCl solution. These plates were grown at 28 °C for 72 h. The diameters of the colonies on each plate were measured for comparison and images of the plates were also taken. Three replicates of each experiment were performed.

## Supplementary Information

**Additional file 1: Fig. S1** The digital pictures of the *T. reesei* RUT-C30 and JNTR5 grown on PDA plates for 5 days. **Fig. S2** The Copy numbers of gene *Tr 69957* in strain JNTR5 **(a)** and the FPKM values of gene *Tr 69957 *in strain RUT-C30 and JNTR5 **b**. **Fig. S3** Photographs taken under 302 nm UV light of the yellow pigments produced by strain JNTR5. The samples were obtained by cultivitating JNTR5 at 28 °C for 96 h with glucose as carbon source. **Fig. S4** Confocal images of *T. reesei* RUT-C30 and JNTR5 observed by fluorescence microscopy by 10 X objective lens at E_x_/E_m_ = 488/498-563 nm. The samples were obtained by cultivitating RUT-C30 and JNTR5 at 28 °C for 48 h with glucose as carbon source. **Fig. S5 **The cellulase/hemicellulase activities of JNTR5. Error bars indicate SDs from three independently cultured replicates. **Table S1** Primers used in this study

**Additional file 2.** Differentially expressed genes (DEGs) based on gene expression profile of T. reesei JNTR5 and RUT-C30.

## Abbreviations

pNPGase: The β-glucosidase activity
pNPCase: The CBH activity
CMCase: The CMC activity
FPase: The filter paper activity
pNPXase: The β-xylosidase activity
AMT: Agrobacterium tumefaciens-mediated transformation
PDA: Potato dextrose agar
SDB: Sabouraud dextrose broth
TMM: Trichoderma minimal medium
MFS: Major facilitator superfamily sugar transporter
