# Supplementary material for: Sorbicillinoids hyperproduction without affecting the cellulosic enzyme production in Trichoderma reesei JNTR5
Source: Biotechnol Biofuels Bioprod. 2022 Aug 22;15:85. doi: 10.1186/s13068-022-02183-1 (PMC9394075; doi:10.1186/s13068-022-02183-1)
Supplement: Supplementary file 1 — Additional file 1: Fig. S1 The digital pictures of the T. reesei RUT-C30 and JNTR5 grown on PDA plates for 5 days. Fig. S2 The Copy numbers of gene Tr 69957 in strain JNTR5 (a) and the FPKM values of gene Tr 69957 in strain RUT-C30 and JNTR5 b. Fig. S3 Photographs taken under 302 nm UV light of the yellow pigments produced by strain JNTR5. The samples were obtained by cultivitating JNTR5 at 28 °C for 96 h with glucose as carbon source. Fig. S4 Confocal images of T. reesei RUT-C30 and JNTR5 observed by fluorescence microscopy by 10 X objective lens at Ex/Em = 488/498-563 nm. The samples were obtained by cultivitating RUT-C30 and JNTR5 at 28 °C for 48 h with glucose as carbon source. Fig. S5 The cellulase/hemicellulase activities of JNTR5. Error bars indicate SDs from three independently cultured replicates. Table S1 Primers used in this study [file 13068_2022_2183_MOESM1_ESM.docx]

**Additional file**

**Sorbicillinoids hyperproduction without affecting the cellulosic enzyme production in *Trichoderma reesei* JNTR5**

Chengcheng Li^1^, Ruihan Gu^1^, Fengming Lin^2,^*, and Huining Xiao^3,^*

^1^ School of Light Ind. & Food Sci. and Jiangsu Co-Innovation Center for Efficient Processing and Utilization of Forest Resources and International Innovation Center for Forest Chemicals and Materials, Nanjing Forestry University, Nanjing 210037, China

^2^ State Key Laboratory of Bioelectronics, School of Biological Science and Medical Engineering, Southeast University, Nanjing 210096, China

^3^ Department of Chemical Engineering, University of New Brunswick, Fredericton, New Brunswick, E3B 5A3 Canada

*Corresponding author:

Fengming Lin, State Key Laboratory of Bioelectronics, School of Biological Science and Medical Engineering, Southeast University, Nanjing 210096, China; E-mail: [linfengming@seu.edu.cn](mailto:linfengming@seu.edu.cn)

Huining Xiao, Department of Chemical Engineering, University of New Brunswick, Fredericton, New Brunswick, E3B 5A3 Canada; E-mail: [hxiao@unb.ca](mailto:hxiao@unb.ca)

**Materials and methods**

**RNA‑seq analysis**

The RNA sequences were processed and analyzed by GENEWIZ according to the reference method. Briefly, total RNA of each sample was extracted using TRIzol Reagent (Invitrogen) Kit (Qiagen) and quantified and qualified by Agilent 2100 Bioanalyzer (Agilent Technologies, Palo Alto, CA, USA), NanoDrop (Thermo Fisher Scientific Inc.) and 1% agrose gel. 1 μg total RNA with RIN value above 7 was used for following library preparation. Next generation sequencing library preparations were constructed according to the manufacturer’s protocol (NEBNext® Ultra™ RNA Library Prep Kit for Illumina®). Illumina RNA sequencing was carried out with two
duplicates by Genscript following their standard analysis method. The sequences of the total reads were mapped to the reference genome of *T. reesei* RUT-C30 from NCBI (https ://www.ncbi.nlm.nih.gov/genom e/323?genom easse mbly_id=49799).


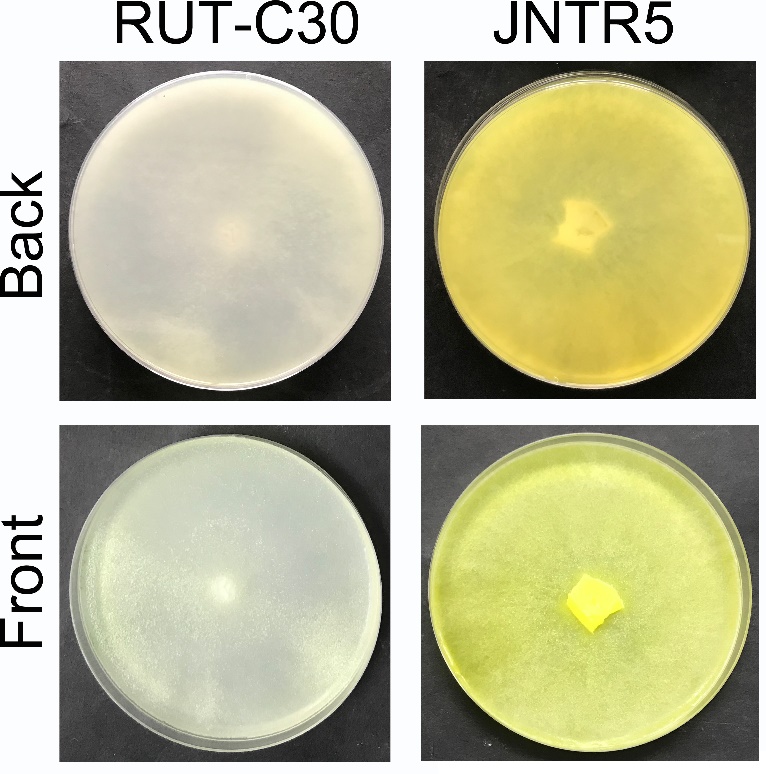


**Fig. S1** The digital pictures of the *T. reesei* RUT-C30 and JNTR5 grown on PDA plates for 5 days.


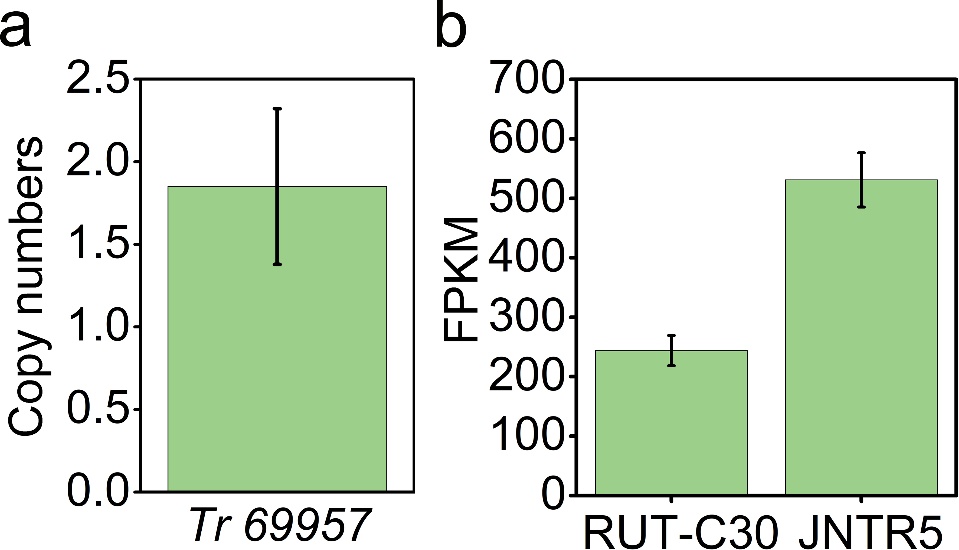


**Fig. S2** The Copy numbers of gene Tr 69957 in strain JNTR5 (a) and the FPKM values of gene *Tr 69957* in strain RUT-C30 and JNTR5 (b).


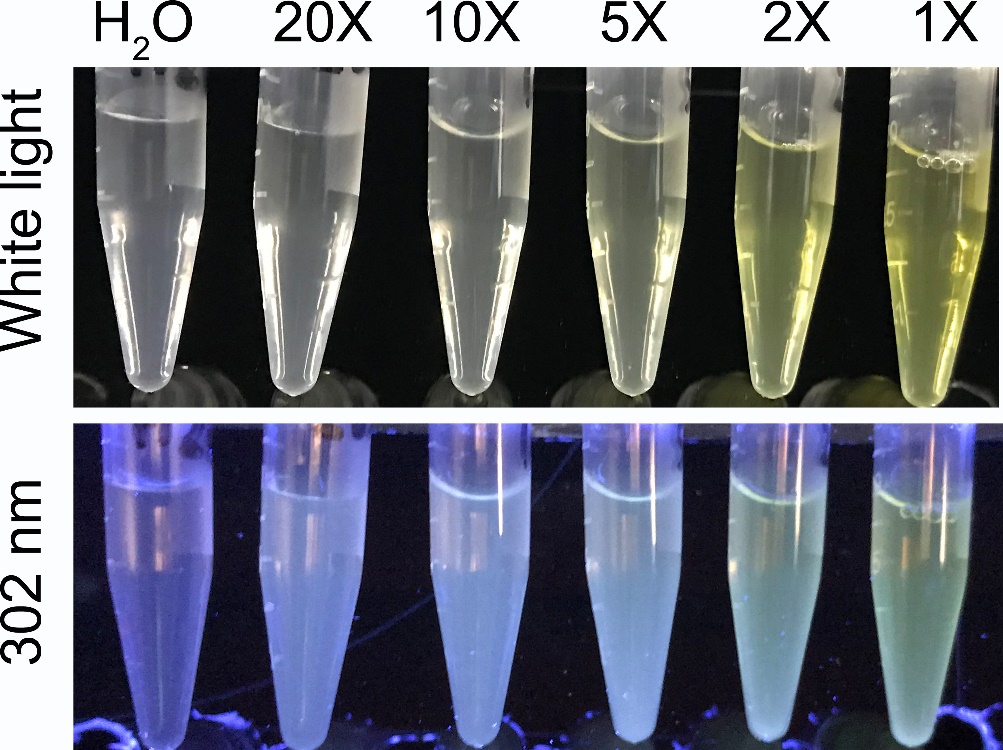


**Fig. S3** Photographs taken under 302 nm UV light of the yellow pigments produced by strain JNTR5. The samples were obtained by cultivitating JNTR5 at 28 ^o^C for 96 h with glucose as carbon source.


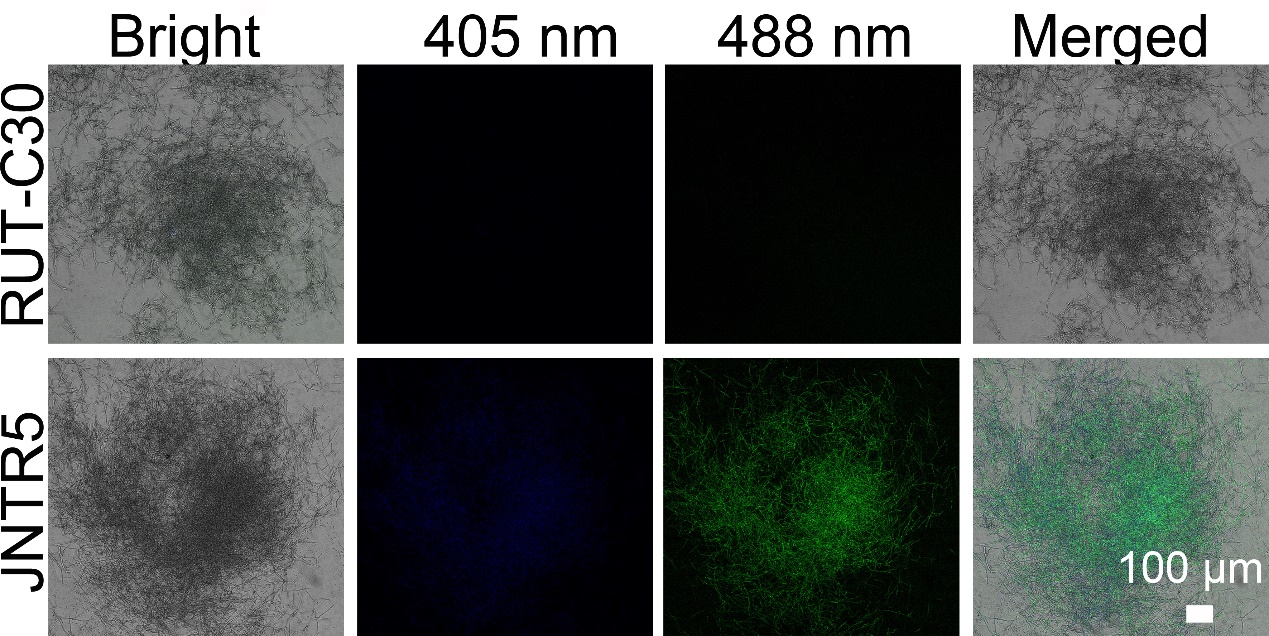


**Fig. S4** Confocal images of *T. reesei* RUT-C30 and JNTR5 observed by fluorescence microscopy by 10 X objective lens at E_x_/E_m_ = 488/498-563 nm. The samples were obtained by cultivitating RUT-C30 and JNTR5 at 28 ^o^C for 48 h with glucose as carbon source.


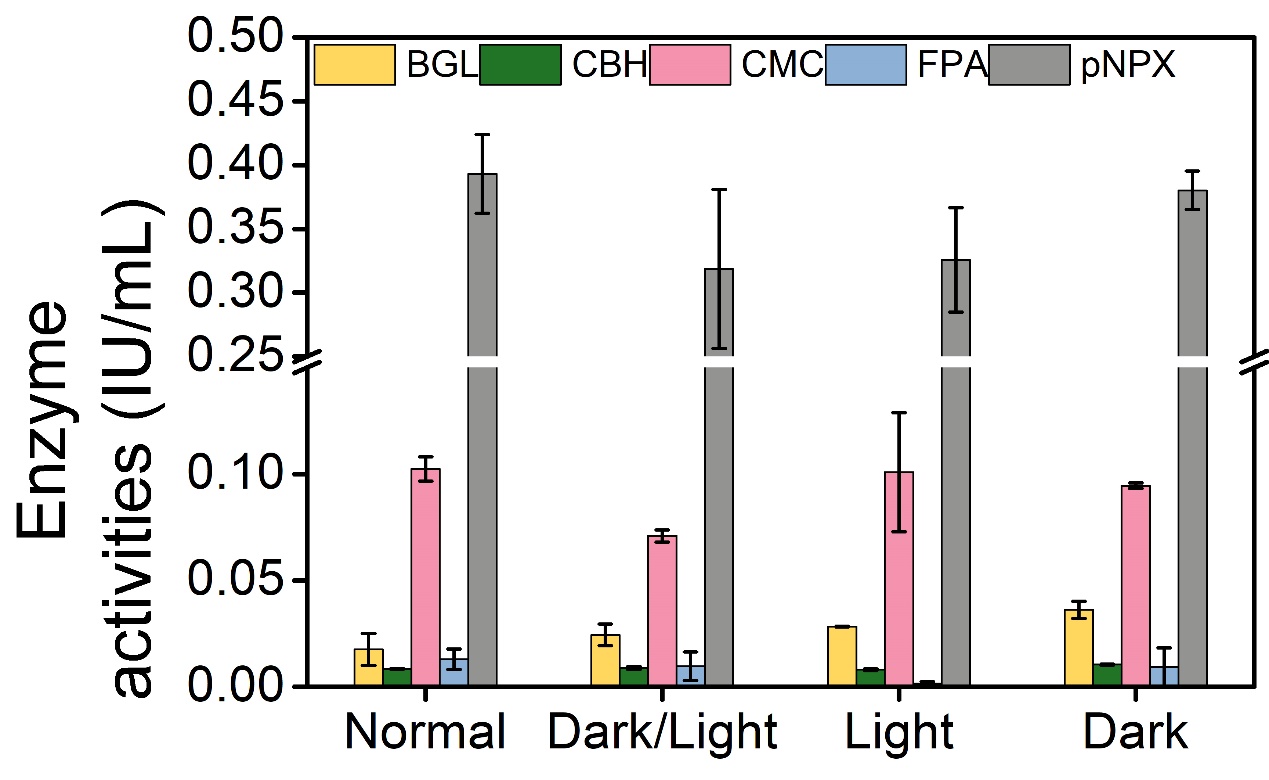


**Fig. S5** The cellulase/hemicellulase activities of JNTR5. Error bars indicate SDs from three independently cultured replicates.

Table S1 Primers used in this study

| Primer name | Sequence |
| --- | --- |
| Tr69957-F | AAACCCAATAGTCAATCTAGAATGGCGGAGGTCAACATCAAGGCT |
| Tr69957-R | CGGTCGGCATCTACTTCTAGATCAAGAAGAATGGGTCTTCTTGGACATGG |

Table S2 Primers for determination of copy numbers of Tr 69957 in JNTR5 using qRT-PCR.

| Primer name | Sequence |
| --- | --- |
| cel7a-C-1-F: | TACCTTATGGCGAGCGACAC |
| cel7a--C-1-R: | GCGTCAGGGGTTCATGGTAA |
| cel7a--C-2-F: | GCCAGGTCCTGAACCCTTAC |
| cel7a--C-2-R: | CCAAGAATCTACCGGTGCGT |
| cel7a--C-3-F: | ACTCCATCTCCGAGGCTCTT |
| cel7a--C-3-R: | CGTCTCGAACTGGGTGACAA |
| sar1-C-1-F: | GCCGACTCTCCACCCTAGTA |
| sar1-C-1-R: | GAAACCCTCCACAAACCCCA |
| sar1-C-2-F: | CCGGAAAGACCACGTTGCTA |
| sar1-C-2-R: | CCTCCTCCCATCGCAGAAAA |
| sar1-C-3-F: | ATTCCCTTTCTCCGTTGCGG |
| sar1-C-3-R: | CCGGATGTCGCATCAGGTAA |
| pgk1-C-1-F: | CCCCAAGTACTCCCTCAAGC |
| pgk1-C-1-R: | GACAATGGACTCGACCTCGG |
| pgk1-C-2-F: | AACCTCCGCTTCCACATTGA |
| pgk1-C-2-R: | GTAGATGTCGCCCAGAGCAG |
| pgk1-C-3-F: | TCATTATCGGCGGTGGTGAC |
| pgk1-C-3-R: | ACCTTTTCGCCATCTCTCCC |
| Tr69957-C-1-F | ATCTCGATTCGGTTACCGGC |
| Tr69957-C-1-R | GGAAGCGTAGGATGGACTCG |
| Tr69957-C-2-F | CCAAGCGAAAGCAAGCAAGT |
| Tr69957-C-2-R | ACCGTTCCGAGAAAAGCGAT |
| Tr69957-C-3-F | CCTGGGGAGTCTTTGCAGTT |
| Tr69957-C-3-R | CAAGTACACCTGCGGCAATG |
